# Reply to Holden and Errington, “Type II Toxin-Antitoxin Systems and Persister Cells”

**DOI:** 10.1128/mBio.01838-18

**Published:** 2018-09-25

**Authors:** Frédéric Goormaghtigh, Nathan Fraikin, Marta Putrinš, Vasili Hauryliuk, Abel Garcia-Pino, Klas Udekwu, Tanel Tenson, Niilo Kaldalu, Laurence Van Melderen

**Affiliations:** a Cellular and Molecular Microbiology (CM2), Faculté des Sciences, Université Libre de Bruxelles (ULB), Gosselies, Belgium; b Institute of Technology, University of Tartu, Tartu, Estonia; c Department of Molecular Biology, Umeå University, Umeå, Sweden; d Laboratory for Molecular Infection Medicine Sweden (MIMS), Umeå University, Umeå, Sweden; e Department of Molecular Biosciences, The Wenner-Gren Institute, Stockholm University, Stockholm, Sweden; National Institute of Child Health and Human Development (NICHD)

**Keywords:** *E. coli*, persisters, toxin-antitoxins

## REPLY

We thank David W. Holden and Jeff Errington for their comments (1). We agree that scientific research is inherently error-prone. Therefore, validation, reassessment, and reinterpretation of one's own and others’ results is part of the scientific approach, regardless of whether the conclusions are affirming or critical. We also agree that further work is needed to establish whether, and in which settings, each individual toxin-antitoxin (TA) system contributes to persister formation in Escherichia coli K-12 and/or in other bacterial species. In the following paragraphs, we express our opinion on the topics on which Holden and Errington saw some overstatements and factual inaccuracies in our paper (2).

The reason we tested the persistence phenotype of the newly constructed Δ10TA strain of E. coli K-12 at mid-exponential growth phase is because the original study by Maisonneuve et al. proposing the link between the TA systems on persister formation was performed under these conditions (3). We cannot therefore draw conclusions about the role of TA systems in E. coli K-12 under other experimental conditions. However, the key outcome of our study and the paper published by Harms and colleagues (4) is a call for setting up all necessary controls, cautiously double-checking new results, and critically reevaluating previously published findings that link TA systems and persister formation.

Holden and Errington use several publications as evidence for TA’s role in persistence. For example, an influential and much-cited study by Harrison et al. reported that deletion of the *yafQ* gene, encoding 1 of the 10 toxin genes deleted in the Δ10TA strain, caused a drop in persister levels surviving cefazolin (a cephalosporin) and tobramycin (an aminoglycoside antibiotic) in E. coli grown as a biofilm (5). To the best of our knowledge, no follow-up or independent study confirming this result has been published. Therefore, we draw attention to several crucial control experiments lacking in the original paper. First, given that the YafQ-inhibiting antitoxin DinJ itself has been reported to affect the general stress response by decreasing RpoS levels (6), it is necessary to assess whether deletion of the full *dinJ-yafQ* operon has the same effect as deletion of the sole *yafQ* gene. Additionally, deletion of *yafQ* had no effect on persistence to doxycycline or rifampin (5), indicating that YafQ does not play a general role in persistence. Second, restoration of the original persister level by complementation of the *dinJ-yafQ* knockout from a plasmid or reinsertion of the operon into the chromosome is essential for drawing reliable conclusions from the phenotypes observed with the knockout strain. We have previously encouraged complementation for validation of the gene knockout effects that change persister levels (7). Third, it is worth mentioning that an independent report showed that deletion of the *dinJ-yafQ* system, as well as other TA systems, did not reduce the persistence of E. coli biofilms in the presence of ofloxacin (8).

Holden and Errington recall accurately that ideas connecting TA systems to persistence go back to an E. coli mutant with enhanced levels of persister formation due to the *hipA7* gain-of-function allele of the *hipA* TA system toxin gene (9). Several other papers recently reported the selection of hyper-persistent mutants of E. coli (10, 11). In addition to mutations in antitoxin genes (*vapB* and *yafN*) (11), mutations in metabolic genes, such as *eno*, *nuoN*, *gadC*, *oppB*, and *pyrG*, as well as mutations in stress response pathways (*rcsD*, *spy*) (10, 11) were selected. First, this tells us that there are multiple pathways that when mutated lead to an increase in persistence, showing that persistence can be attributed to many different mechanisms and supporting the hypothesis that persistence is not an evolved character but rather the “inadvertent product of different kinds of error and glitches” as formulated by Levin and colleagues (12). Second, the mutant phenotype is not necessarily informative about the biological function of the gene, and we should avoid concluding that these genes, whether TA genes or others, were selected by evolution for persistence. Regarding the observation that overexpression of toxin-encoding genes increases persistence, Holden and Errington themselves cite an important and often misinterpreted study by Vázquez-Laslop et al., who specifically questioned the validity of overexpression experiments (13). They have shown that not only TA toxins but also other proteins which stop bacterial growth upon overexpression induce persistence. A similar increase in persistence is observed upon pretreatment of bacterial cultures with bacteriostatic antibiotics (14). Since induction of antibiotic tolerance is evidently a common effect of bacterial growth inhibition, we strongly believe that experiments relying on overexpression of a toxin or gain-of-function alleles of TA systems do not constitute evidence for TA systems being causative agents of the naturally occurring persister phenotype.

In conclusion, we are sure that both the mechanisms of antibiotic persistence and the functions of individual TA systems deserve further rigorous, careful, and controlled study if we are to maintain high standards of scientific quality.
